# COVID-19 and End of Life in a Quaternary Australian Hospital: Referral for Palliative Care Consultation

**DOI:** 10.1089/pmr.2023.0069

**Published:** 2024-01-05

**Authors:** Chelsea Patterson, Linda Foreman

**Affiliations:** Central Adelaide Palliative Care Services, Adelaide, South Australia, Australia.

**Keywords:** COVID-19, palliative care consultation, prescription patterns, referral

## Abstract

**Background:**

The coronavirus disease 2019 (COVID-19) pandemic resulted in complex physical and psychosocial symptom burden at end of life. The benefit of specialist palliative care input in other disease states has been established, however, there is little evidence on referral patterns to these services in patients dying from COVID-19.

**Objectives:**

This retrospective audit investigated the referral patterns for patients who died from COVID-19 at a quaternary hospital in South Australia (the Royal Adelaide Hospital) over a six-month period in 2022, and whether demographic features or COVID-19 specific factors had an impact on whether these patients received specialist palliative care services (PCS). The second aim was to identify prescription patterns for patients in the last 24 hours of life, and whether this was impacted by referral.

**Method:**

Data were obtained from electronic medical records and analyzed using binary logistic regressions for referral to PCS versus no referral based on various predictors.

**Results:**

There was no significant difference comparing patient demographics or COVID-19 specific factors with referral to PCS. There was statistical significance between patients who received referral to PCS and those who had a higher oral morphine equivalent daily dose (OMEDD) in the 24 hours before death, as well as the presence of a continuous subcutaneous infusion. Although the cause of this relationship is undetermined, it may represent the prescription patterns of the palliative care physicians during consultation or potentially higher symptom burden prompting referral. There was also a higher proportion of patients who received hydromorphone compared with other opioids, though the OMEDD was consistent with other published literature.

## Introduction

The coronavirus disease 2019 (COVID-19) pandemic has presented new challenges globally within health care services. With significant morbidity and mortality, COVID-19 has caused 767 million cases of infection worldwide, and 6.9 million cumulative deaths.^1^ Despite rapid development of preventative vaccinations and evolving therapies providing survival benefit, patients admitted to hospital still have a significant mortality risk, particularly those who are elderly or medically comorbid.^2,3^

Palliative care input has been associated with improved quality of life, symptom management, and reduced caregiver distress in patients with malignant and nonmalignant terminal disease.^4–7^ This pandemic adds challenges that increase the prevalence of complex physical and psychosocial needs, including risks of rapid respiratory deterioration, isolation and restricted family visits, and limitations in health care resources.^8^

Access to specialist palliative care health care services has been recognized as an important component of caring for patients during the pandemic.^9^ Despite this, there are no evidence or consensus-based guidelines regarding when specialist input is recommended, or which patients obtain the most benefit from this input. Referrals to specialist palliative care teams are affected by patient, physician, disease, and service factors, and the impact of COVID-19 on these referrals is unknown.

Owing to public health measures at a national and state-wide level within the state of South Australia (SA), the local health system saw a delayed onset to high number of infections requiring admission to the public hospitals compared with other national and international locations.^10,11^ This occurred in early 2022, following relaxation of border restrictions. The Royal Adelaide Hospital (RAH) is a quaternary center that was designated as the primary hospital within SA for COVID-19-infected patients. A palliative care consult liaison (PCCL) service is available at the RAH, receiving referrals from inpatient teams, and comprising palliative care consultants, registrars, nurse consultants, and social workers.

Once referred to PCCL, the patient's care is guided by the palliative care recommendations (particularly regarding medication prescription), however, the primary care team remains involved with the goal of optimizing continuity and therapeutic relationships. The primary aim of this study was to identify whether there were specific factors that influenced referral by comparing characteristics of those who died from COVID-19 with or without a referral to the RAH PCCL service during their admission. The secondary aim was to contribute descriptive statistics to the end-of-life pharmacological management of patients dying with COVID-19.

## Methodology

### Design and setting

This was a retrospective audit of patients who died in the RAH with COVID-19 between January 1, 2022, to June 30, 2022. The RAH was the primary center of care for COVID-19-infected adult patients for the duration of the study.

For the six-month period, most patients who were admitted with COVID-19 infections were allocated to a specifically created COVID-19 general medicine unit. This included patients whose care solely occurred in the intensive care unit, or the emergency department. At the end of the six-month period, a list of inpatient deaths from the COVID-19 general medicine unit was obtained. This audit did not include patients who were infected with COVID-19 during their inpatient admission under the care of another team or those who had incidental COVID-19 infection with another primary pathology requiring hospital admission and alternative specialist input.

Patients who died while in the intensive care unit were excluded, as their end-of-life care was managed through a separate structure. A list of all COVID-19-infected patients who were referred to the PCCL was also maintained throughout this period. The endpoint of “death” was selected to ensure identification of an objective population to study.

### Ethics approval

Ethics approval was initially obtained on June 22, 2022, through the Central Adelaide Local Health Network Human Research Ethics Committee, and approved for publication on August 25, 2023 (reference number 18373).

### Data collection and management

The electronic medical records of 149 patients who died with COVID-19 during the described period were reviewed. Data were extracted from administrative, medical, and nursing documentation, and medication administration charts. Demographic characteristics including age, gender, country of birth, and place of residence (independent home, residential aged care facility [RACF], or disability supported care) were recorded, as were the patients' Charlson comorbidity index (CCI) scores before admission.

For those referred to PCCL service, an Australia-modified Karnofsky performance status (AKPS) and palliative care phase (stable, unstable, deteriorating, or terminal) were recorded at the time of first contact. The patient location (general ward or emergency department) and the referrer role were also noted. The reason for referral was also extracted, if documented, as was presence or absence of specific therapies (use of continuous subcutaneous infusion [CSCI] for symptom management, or high-flow nasal cannula for oxygen delivery on the ward). The date of onset of COVID-19 infection and subsequent time to referral to PCCL (if applicable) and death were also recorded.

### Statistical methods

Binary logistic regressions were performed for the outcomes of referral to PCCL versus no referral to PCCL based on various predictors. Results (odds ratios, 95% confidence intervals [CIs] and *p*-values) are presented below. The statistical software used was SAS On Demand for Academics (SAS Institute Inc., 2021). A *p*-value <0.05 was considered statistically significant.

## Results

From January 1, 2022, to June 30, 2022, there were 149 patients who died while admitted under the specified COVID-19 general medicine unit. Of these, six were admitted under this team with presumed COVID-19 infection, but this was subsequently excluded on formal testing. These patients were excluded, as were two patients whose electronic medical records were sealed due to ongoing coronial investigation.

Of the remaining patients, 101 were admitted in a medical ward or in the emergency department (rather than the intensive care unit) at time of death. This was the final number of included patients. Demographic information is given in Table 1. Twenty-eight (27.7%) of these patients were referred to the PCCL team.

**Table 1.** Demographic Information of Patient Groups

	Referred to PCCL	Not referred to PCCL	Total
Age (average, years)	80.6	84.1	83.1
Gender (male, %)	60.7	55.4	53.4
Location before admission (total, %)			
Home	12 (42.9)	22 (30.1)	34 (33.7)
RACF	15 (53.6)	49 (67.1)	64 (63.4)
NDIS supported living	1 (3.6)	2 (2.7)	3 (3)
CCI (average)	6.4	6.7	6.6
Country of birth (total, %)			
Australia	13 (46.4)	34 (46.6)	47 (46.5)
International	15 (53.6)	39 (53.4)	54 (53.5)
Known to palliative care service before admission (total, %)	4 (14.3)	6 (8.2)	10 (9.9)

CCI, Charlson comorbidity index; NDIS, National Disability Insurance Scheme; PCCL, palliative care consult liaison; RACF, residential aged care facility.

The average age of the population was 83.1 years, with slightly higher proportion of male patients (53.4%). There is not a statistically significant association between referral group and age (*p* = 0.1426). Most patients were residing in a RACF before admission (63.4% total), with no statistically significant difference between location before admission and referral to PCCL (*p* = 0.2138).

The average CCI was 6.6, reflecting a highly comorbid population with significant in-hospital mortality risk. The number of patients known to palliative care services (PCS) before admission was small—only 10 patients (9.9%). Four of these patients were subsequently re-referred during their admission for inpatient PCCL input, and the remaining six were not. There was no statistical significance with either CCI or prior palliative care input and referral to PCCL.

At the time of documented change to direction of care (to a symptom-based or palliative approach to care), 28 patients (27.7%) were receiving oxygen through high-flow nasal cannula (HFNC). This was similar whether the patients were referred or not (28.6% and 27.4% respectively, *p* = 0.9061). It was not recorded whether this high-flow oxygen delivery method was weaned off before death.

The average length of time from COVID-19 diagnosis to death varied, with an average length of 12.3 days. There was a trend to longer length of time in patients referred to PCCL (15.6 days) compared with those who did not (11 days), though this was not statistically significant (*p* = 0.0862). The range of time from referral to death varied—between 0 (referral on the day of death) to 31 days, with an average of 4.9 days. Most of the patients referred were seen face to face, however, three patients were referred and received input only in the form of phone advice to the referrer. These patients died the day of contact. Of those seen face to face (25 patients), the number of unique encounters with PCCL ranged from 1 to 11 (mean 3, median 2). Postdeath bereavement follow-up (to the appropriate next-of-kin by phone call) was subsequently completed by a palliative care social worker in 60% of these cases.

Nearly half the patients received medication through a CSCI in the last 24 hours of life (48.5%). There is a statistically significant association between the referral group and having a CSCI (*p* = 0.0001). Of those referred to PCCL, 23 (82%) received medicine through a CSCI, which was commenced after the involvement of PCCL in the majority (20) of cases. Twenty-six (35.6%) of patients not referred to PCCL were also commenced on a CSCI.

Medication prescription patterns in CSCIs are documented in Table 3. The most common opioid administered through CSCI was hydromorphone (33, 67%) followed by morphine (15, 30.6%). One patient received fentanyl. There is a statistically significant association between oral morphine equivalent daily dose (OMEDD) 24 hours before death and CSCI (*p* < 0.0001). Those patients with CSCI had a mean OMEDD value 59.6 mg greater than those patients without CSCI (mean difference = 59.6, 95% CI: 46.6–72.6).

**Table 2.** Binary Logistic Regressions of Referral Group Versus Predictors

Predictor	Comparison	OR^a^	95% CI	Comparison ***p***-value	Global ***p***-value
Gender	Female vs. male	0.74	(0.31–1.80)		0.5100
Age	Per 1-year increase	0.97	(0.93–1.01)		0.1426
Country of birth	Australia vs. international	0.99	(0.42–2.38)		0.9894
Home location	Home vs. NDIS provided housing	1.09	(0.09–13.31)	0.9456	0.4519
Home location	Home vs. RACF	1.78	(0.72–4.43)	0.2138	
Home location	NDIS provided housing vs. RACF	1.63	(0.14–19.29)	0.6969	
CCI	Per 1 unit increase	0.92	(0.73–1.16)		0.4924
Known to palliative care service before admission	Yes vs. no	1.86	(0.48–7.17)		0.3666
CSCI commenced	Yes vs. no	8.32	(2.83–24.47)		0.0001
Requiring high-flow nasal cannula at the time of change in goals of care	Yes vs. no	1.06	(0.40–2.79)		0.9061

^a^ Modeling the probability of being referred to palliative care.

CI, confidence interval; CSCI, continuous subcutaneous infusion; OR, odds ratio.

**Table 3.** Medication Prescription Patterns in Continuous Subcutaneous Infusions

	Referred	Not referred	Total
Opioid (total, %)			
Hydromorphone	15 (65.2)	18 (69.2)	33 (67.3)
Morphine	7 (30.4)	8 (30.8)	15 (30.6)
Fentanyl	1 (4.3)	0	1 (2)
Benzodiazepine (total, %)			
Midazolam	20 (87)	19 (73.1)	39 (79.6)
Clonazepam	0	1 (3.8)	1 (2)
Antisecretory (total, %)			
Hyoscine Butylbromide	7 (30.4)	8 (30.8)	15 (30.6)
Antipsychotic (total, %)			
Haloperidol	2 (8.7)	1 (3.8)	3 (6.1)

There is also a statistically significant association between OMEDD 24 hours before death and referral to PCCL (*p* = 0.0001). Those patients who were referred had a mean OMEDD value 35.3 mg greater than those patients not referred (mean difference = 35.3, 95% CI: 17.1–53.5).

The documented indication for referral was often brief, or not clearly stated. When documented, the most common listed reason was for “assistance with end-of-life care,” without specification of what triggered request for specialist input (Table 4). There was no AKPS higher than 50, with most patients either considered comatose (AKPS 10) or totally bedfast and requiring extensive nursing care (AKPS 20).

**Table 4.** Palliative Care Consultation Referral Descriptors

	Total, ***n*** (%)
Documented indication for referral	
Assistance with end-of-life care	14 (50)
Symptom management	10 (35.7)
Discharge planning	2 (7.1)
Australian-modified Karnofsky performance status (at time of first PCCL review)	
10	12 (42.9)
20	13 (46.4)
30	2 (7.1)
40	1 (3.6)
>50	0
Method of contact	
Face to face	25 (89.2)
Phone advice	3 (10.7)

## Discussion

The demographic profile of patients included in this study was similar to those seen internationally in patients dying from COVID-19, including the high prevalence of comorbidities, with the average CCI of 6.6 well aligned with other studies.^12–14^ The majority (63%) lived in RACF before their admission, reflecting this degree of comorbidity. The percentage of patients born overseas was higher than the national average (53.5% compared with 30%), however, this is likely to be related to the older age group included and Australia's historical pattern of immigration.^15^

None of these individual patient factors before admission were associated with any difference in rate of referral. Although there was also no relationship identified between whether patients were known to PCS before referral and whether they were referred, the number of patients in this category was too low to infer significance.

The average OMEDD in a CSCI was 57 mg, with hydromorphone the most common opioid prescribed whether they were referred to PCCL or not. In those referred to PCCL, this opioid choice was guided by the consulting service, and by the primary care team in those not referred. The prevalence of hydromorphone use most likely reflects local practice and is notably different from other published opioid prescription patterns in patients dying with COVID-19.^12,16^ The use of hydromorphone in this audit was interestingly not consistently associated with impaired renal function, which is one of the indications specified on local prescribing guidelines for hydromorphone as the opioid of choice in end-of-life care, though this remains debateable in the literature with a lack of consistent evidence supporting this recommendation.^17–19^

The presence of hydromorphone in these local guidelines is likely to have increased knowledge of this opioid as an option, and contributed to the pattern seen, even without the presence of established renal failure. The indication for opioid use is presumed to be primarily dyspnea due to the underlying respiratory illness rather than pain based on other published data on symptom burden in patients dying with COVID-19, though data on indication were not collected in this study.^20,21^

The average subcutaneous morphine equivalent in a CSCI was 19 mg, which is consistent with other published doses seen worldwide in similar patient cohorts.^12,16^ The total average OMEDD including *Pro Re Nata* (PRN) medication was 61 mg, with a significantly higher OMEDD in patients referred to PCCL compared with those not (79.2 and 43.9 mg, respectively, *p* < 0.0001), and a higher proportion who were then managed with medication administered through CSCI. This could imply that there is identification of patients with distressing physical symptoms who may warrant specialist palliative care input, though it does not reflect or examine whether severe patient or family psychological distress was also a factor.

An alternative explanation is that specialist palliative care teams are more familiar with the use of CSCIs to manage symptoms, and more comfortable prescribing higher doses of opioids based on experience and the interpretation of patient distress. The average length of time between referral to death was 4.9 days, which would allow for repeated review and increase of opioids by the palliative care team. A 2021 Australian study from the state of Victoria found that 71% of COVID-19 patients referred for end-of-life symptom management were commenced on a CSCI by the palliative care team, which is the same percentage in this study.^22^ By contrast, only 35.6% of patients who did not have PCCL involvement were commenced on a CSCI.

There is little published literature about referral to palliative care for patients with COVID-19. Given the severity of illness, patients with COVID-19 would benefit from specialized palliative care input focusing on complex decision making and care needs, including physical, psychosocial, and spiritual.^8,9^ A Canadian qualitative review explored potential barriers to referral. Whether the referrer considered COVID-19 as a “palliative diagnosis,” as well as uncertainty about disease trajectories with new rapidly emerging therapies, was cited as COVID-19 specific barriers to referral in this study.^23^

Institutional or unit-based knowledge and receptiveness to palliative care has been documented to be a barrier to integration particularly in nononcological attempted to mitigate this by selecting patients admitted under one general medicine unit, however, within this unit there were separate medical teams comprising consultants, registrars, resident medical officers, and interns, all of whom had different experience with palliative medicine or end-of-life care. In addition, although there are local prescribing guidelines that provide pharmacological management advice for end-of-life care, the awareness of these guidelines among physicians is variable.

These physician factors contributing to referral are difficult to quantify without more structured qualitative analysis and were not captured in this study. As only physicians can refer for PCCL involvement at this hospital, this reduces the chance for other care providers to recognize and action a need for referral.

Previously studied populations experiencing pandemics (primarily in Africa or Asia) have identified access to PCS (including shortages in equipment, staff, and medication) as a significant barrier to referral.^23,25,26^ Despite this study being performed during the initial peak of COVID-19 infections in SA, inpatient access to PCS was not limited by staffing. All patients referred received specialist advice, with a small minority receiving this over the phone due to rapid clinical deterioration, and medication prescription choice and delivery were not impacted by availability. This ready response of the PCCL team may have increased awareness of the service and overall increased the referral rate, though this was not directly measured.

### Limitations

The number of patients included in this audit was relatively small and was impacted by patient selection and the length of time studied. A specific exclusion was those patients who died in the intensive care unit. This was decided as these patients had significantly different end-of-life management than those in the wards—notably the presence of invasive ventilation, central intravenous access, and propensity to prescribe opioids in a different manner, when compared with CSCIs and subcutaneous PRN medication, which impacted direct comparison. However, this did exclude a third of the patients who died during the period studied. This contributed to the study being underpowered to assess certain factors that may have influenced referral (i.e., those who were already known to PCS).

The design of this retrospective audit relied in part on accurate documentation from the treating medical teams involved, with the lack of a standardized symptom assessment and limited interpretation of indication for referral. For example, the documented reasons for referral to PCCL were not specific and did not elucidate further information about symptom burden. A quantitative review of medication used was also insufficient to determine the indication for use, and thus infer symptom burden. Benzodiazepines were used in 79.6% of patients, however, this may have been used for multiple reasons, including dyspnea, anxiety, or perceived distress.

COVID-19 patients have a high prevalence of psychological distress, including anxiety, depression, and disturbed sleep.^20,27,28^ Suffering can be exacerbated by social isolation and fear of deteriorating or dying alone. This can be particularly poorly represented by record of medication use. In addition, there were factors that could have further influenced medication prescriptions that were not assessed, such as whether patients who were receiving oxygen through HFNC had this weaned before death. There is scope for exploration of this in future research.

This was also a single-center study, and as discussed above, there are local practice guidelines that have a large impact on prescribing and referral patterns.^19^ This was observed not only within the PCCL team but also the entire studied population, as evidenced by the prevalence of hydromorphone use irrespective of PCCL involvement in comparison with other worldwide published data.

## Conclusion

This study has demonstrated that the patients dying from COVID-19 who were referred to the PCCL service were referred irrespective of their background demographics, comorbidities, prior exposure to palliative care, or the presence of noninvasive ventilation. Those who were referred to palliative care were more likely to have a higher opioid medication use and more likely to be commenced on a CSCI. This is likely to represent experience with prescribing end-of-life medication by the palliative care team, but potentially could be contributed to by recognition of higher symptom burden resulting in referral (though this was not able to be concluded within the limitations of this study).

It has also added to the published literature of descriptions of prescription patterns for end-of-life care in COVID-19-infected patients in a quaternary Australian hospital, particularly noting a predilection toward the use of hydromorphone that has not been observed in other literature.

Given that the documented physical, psychological, and social complexities associated with dying during a pandemic are especially difficult to assess quantitatively with medication use, it would be prudent for further prospective qualitative research to explore whether these patients are receiving referrals appropriately to specialist palliative care units. Ultimately, identification of patients with complex needs and potential factors influencing referral would allow this to be addressed, ensuring patients who need specialist input are receiving it.

## Abbreviations Used

AKPS: Australia-modified Karnofsky performance status
CCI: Charlson comorbidity index
CI: confidence interval
COVID-19: coronavirus disease 2019
CSCI: continuous subcutaneous infusion
HFNC: high-flow nasal cannula
NDIS: National Disability Insurance Scheme
OMEDD: oral morphine equivalent daily dose
OR: odds ratio
PCCL: palliative care consult liaison
PCS: palliative care services
PRN: *Pro Re Nata*
RACF: residential aged care facility
RAH: Royal Adelaide Hospital
SA: South Australia
